# Optimization and Validation of Methods for Mapping of the Radiofrequency Transmit Field at 3T

**DOI:** 10.1002/mrm.22421

**Published:** 2010-07

**Authors:** Antoine Lutti, Chloe Hutton, Jürgen Finsterbusch, Gunther Helms, Nikolaus Weiskopf

**Affiliations:** 1 Wellcome Trust Centre for Neuroimaging, UCL Institute of Neurology, University College LondonUK; 2 Department of Systems Neuroscience, University Medical Center Hamburg-EppendorfHamburg, Germany; 3 MR-Research in Neurology and Psychiatry, University Medical Center GöttingenGottingen, Germany

**Keywords:** RF mapping, quantitative MRI, flip-angle correction, *B*^+^_1_, *B*_1_

## Abstract

MRI techniques such as quantitative imaging and parallel transmit require precise knowledge of the radio-frequency transmit field (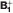). Three published methods were optimized for robust 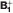 mapping at 3T in the human brain: three-dimensional (3D) actual flip angle imaging (AFI), 3D echo-planar imaging (EPI), and two-dimensional (2D) stimulated echo acquisition mode (STEAM). We performed a comprehensive comparison of the methods, focusing on artifacts, reproducibility, and accuracy compared to a reference 2D double angle method. For the 3D AFI method, the addition of flow-compensated gradients for diffusion damping reduced the level of physiological artifacts and improved spoiling of transverse coherences. Correction of susceptibility-induced artifacts alleviated image distortions and improved the accuracy of the 3D EPI imaging method. For the 2D STEAM method, averaging over multiple acquisitions reduced the impact of physiological noise and a new calibration method enhanced the accuracy of the 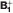 maps. After optimization, all methods yielded low noise 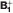 maps (below 2 percentage units), of the nominal flip angle value (p.u.) with a systematic bias less than 5 p.u. units. Full brain coverage was obtained in less than 5 min. The 3D AFI method required minimal postprocessing and showed little sensitivity to off-resonance and physiological effects. The 3D EPI method showed the highest level of reproducibility. The 2D STEAM method was the most time-efficient technique. Magn Reson Med, 2010. © 2010 Wiley-Liss, Inc.

Spatial inhomogeneities of the positive circularly polarized component (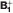) of radiofrequency (RF) *B*_1_ result in the local deviations of the flip angle from its nominal value. These deviations affect quantitative MRI techniques such as perfusion (1), magnetization transfer ratio (2), and *T*_1_, *T*_2_ (3) imaging. In standard MRI, 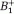 inhomogeneities lead to degradation of image contrast and uniformity, which limit the robustness of image processing techniques such as segmentation even after compensation for RF inhomogeneities has been applied (4–7). 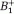 inhomogeneities increase with the strength of the static magnetic field (*B*_0_) due to the corresponding decrease in RF wavelength (8). 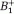 inhomogeneities can be reduced using phased-array transmit coils when the local magnitude and phase of the RF field are known (9,10). This can be achieved by accurate 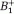 mapping techniques (3,11). For most applications, it is sufficient to know the magnitude |
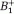
| of 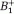. For brevity, we will use the two terms synonymously in the following.

Robust 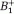 mapping methods are often based on the double angle method (DAM) (12,13), where local flip angle values are estimated from the ratio of two images obtained for different nominal flip angle values. Here, incomplete longitudinal relaxation may bias the resulting flip angle maps for short pulse repetition times (TR) (13). Although compensating pulses (14) and magnetization resets (15) have been introduced in order to allow short TR acquisitions, the DAM method has mainly been implemented with *TR* ≥ 5 × *T*_1_ in order to avoid bias due to longitudinal relaxation, resulting in long acquisition times impractical for routine in vivo applications. In order to speed up whole-brain 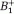 mapping, the DAM method can also be implemented as a multislice technique. However, for two-dimensional (2D) acquisition schemes, 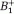 maps are affected by inhomogeneous spin excitation across the slice (16), in-flow artifacts and through-plane blood flow. Nonlinearities between the slice profile and the nominal flip angle lead to misestimated 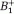 maps and require accurate calibration of the relation between signal (integrated across the slice profile) and pulse amplitude (14,17,18). To overcome these problems, three-dimensional (3D) multishot methods using nonselective excitation have been introduced (19). These methods exhibit higher signal-to-noise ratios and reduced sensitivity to in-flow artifacts but increased sensitivity to motion artifacts (20).

In this work, we focused on in vivo mapping of the magnitude of 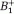 at 3T. If the phase and magnitude of 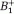 are required, a combination of one of the methods presented here with a fast phase mapping method based on gradient echo acquisitions can be used (21). For routine use in neuroimaging, short acquisition time (<5 min), whole-head coverage, high precision, and accuracy are desirable. From the literature, we selected three 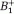 mapping methods that offer high time efficiency: (1) Yarnykh (22) introduced the 3D actual flip angle imaging (AFI) method based on successive fast low-angle-shot (FLASH) acquisitions at interleaved TRs that suffers from minimal spatial distortions and needs one data acquisition only. (2) Jiru and Klose obtained flip angle maps from spin-echo (SE) and stimulated echo (STE) images recorded with a 3D echo-planar imaging (EPI) scheme, allowing for very fast and efficient 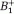 mapping (23). (3) Helms et al. developed a 2D stimulated echo acquisition mode (STEAM) method based on two acquisitions with two different flip-back angles that shows minimal distortions and a high efficiency. Each method was validated on individual subjects and phantoms in the original papers (18). However, 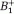 maps were obtained at different field strengths and on different phantoms, so that any comparison between the results would be invalid. Here, we performed a multisubject comparison between the methods to assess their relative advantages, accuracy, and precision. We first optimized the three 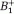 mapping methods for implementation at 3T. The accuracy of each technique was tested against results obtained using a modified version of the robust 2D DAM method proposed by Sled and Pike (24).

## THEORY AND OPTIMIZATION

Here, we briefly review each of the methods and describe the optimizations that we performed. The exact details of the implementation are described in the Materials and Methods section.

### 3D AFI Method

The 3D AFI method records signal using two interleaved FLASH acquisitions with repetition times *TR*_1_ and *TR*_2_ (22). If longitudinal relaxation of the magnetization between the repetitions can be linearized (*TR*_1_ < *TR*_2_ ≪ *T*_1_), flip angle (α) maps can be extracted from the two resulting images using (22):


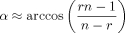
[1]

where *n* = *TR*_2_/*TR*_1_, *r* = *S*_2_/*S*_1_ and *S*_1_, *S*_*2*_ are the signals acquired during *TR*_1_ and *TR*_2_, respectively. Spoiler gradients and RF spoiling as proposed by Nehrke (25) are applied in order to achieve spoiling of the transverse coherences between the gradient echoes.

For further improvement to previous 3D AFI implementations, we introduced flow-compensated diffusion-weighting spoiler gradients in order to improve spoiling of the transverse coherences by diffusion damping (25,26) while keeping the level of physiologic artifacts minimal in the images.

### 3D EPI Method

With the method introduced by Akoka et al. (27), the local flip angles α are extracted from SE and STE signals. In the implementation of this method by Jiru and Klose (23), nonselective RF pulses and 3D multishot EPI acquisitions are used and local flip angles are obtained according to:


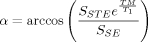
[2]

where *S*_*SE*_ and *S*_*STE*_ are the intensities of the SE and STE images and *TM* is the “mixing time,” i.e., the time span between the spin and stimulated echoes. As local flip angle calculations are based on magnitude images, two possible values of α, i.e., 90 + δ and 90 − δ, satisfy Eq. 2 at each voxel. The correct flip-angle values can be extracted from the repetition of the image acquisition for different values of the nominal flip angle, by a linear regression of nominal versus local flip angles (23).

**FIG. 1:**
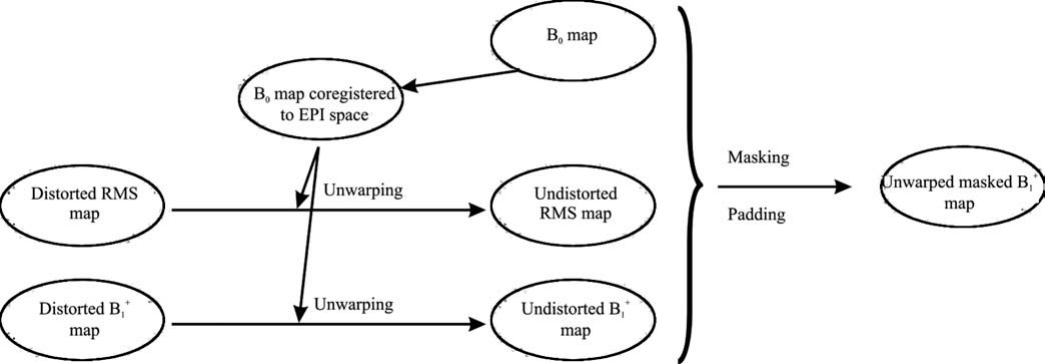
Postprocessing method used for the 3D EPI acquisition scheme. Unwarping of the 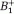 maps is followed by masking based on the local *B*_0_ value and errors on the flip angle estimates (RMS map); 71 × 24 mm (600 × 600 DPI).

In order to increase the robustness of the original method, we developed an image processing method, illustrated in Fig. 1. We used a *B*_0_ map (acquired using a double gradient-echo FLASH acquisition) to correct susceptibility-induced geometric distortions of the 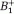 maps (based on EPI acquisitions) following the method described in Hutton et al. (28). Following the linear regression described above, the square root of the residual mean square (RMS) of the fit was calculated at each voxel and divided by the number of nominal flip angle values to provide a map of error values (RMS map). Distortions were also corrected in the RMS maps, which were then used to identify voxels where a poor fit of the linear regression was present. These voxels were masked out of the 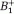 maps, and the missing flip angle values were estimated by averaging those of the remaining neighboring voxels (*RMS padding*). Preliminary experiments showed that *B*_0_ inhomogeneities could affect spin precession due to off-resonance effects, altering the measured effective flip angle. Padding was therefore also applied on the 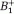 maps at voxels where the local *B*_0_ field exceeded a threshold value (determined by the bandwidth of the RF pulses used in our experiment) (*B*_0_ *padding*). The masks based on the RMS and *B*_0_ values were effectively combined using an inclusive “or” operation before performing the padding.

### 2D STEAM Method

This rapid method is based on multislice single-shot STEAM MRI exploiting the signal dependence on the flip angle of the second (“flip back”) pulse (18). Relative deviations of the flip angle from the nominal value are given by the spatially dependent factor *f*_*T*_ according to:


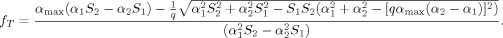
[3]

Here *S*_1_ and *S*_2_ are image intensities obtained for two nominal flip angles α_1_ and α_2_ of the “flip-back” pulse. This equation already comprises a transformation from the slice-selective signals to nonselective excitation. Equation 3 is based on a quadratic approximation of the sinusoidal flip angle dependence around its maximum:


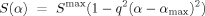
[4]

where α_max_ and *q* are pulse-shape-dependent calibration parameters accounting for nonlinearities (shift and width) of the slice profile as the nominal flip angle is varied (18). In the original calibration method, α_max_ and *q* are determined by comparison to a nonselective flip-back pulse in a separate fully relaxed experiment on a phantom.

In this work, calibration of the nonlinearities of the slice-selective pulse was implemented by minimizing deviations between the 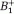 maps obtained using the 2D STEAM and 3D AFI methods on a head-sized gel phantom. This calibration procedure was performed only once prior to in vivo scanning and the whole central volume of the phantom was used (instead of a small central region of interest [ROI], as described in Helms et al. (18)). For in vivo scanning, three consecutively acquired 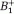 maps were averaged in order to reduce instabilities due to the spurious occurrence of postsystolic cerebrospinal fluid flow through the image slices and head movements. As a result, one single 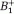 map was produced with a reduced level of physiologic artifacts.

### Reference 2D DAM Method

A reference 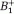 map was acquired on each subject based on the application of a nonselective presaturation RF pulse followed by a slice-selective excitation pulse, similarly to the (saturation) DAM method introduced by Sled and Pike (24). Flip angle maps were calculated from the image intensities (*S*_1_ and *S*_2_) obtained for two values α_1_ and α_2_ of the presaturation flip angle:


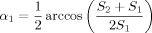
[5]

with α_2_ = 3×α_1_. Signal was acquired using a multislice 2D EPI readout in order to reduce the scan time. Geometric distortions of the DAM 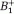 maps were corrected using the unwarping procedure described in Fig. 1 for the 3D EPI method. Since the local flip angles were derived from variations of the nonselective presaturation pulse, any errors associated with slice selection were avoided. Complete relaxation of the magnetization was allowed between each repetition (i.e., *TR* ≥ 5 × *T*_1_). The 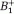 maps obtained from the 3D AFI, 3D EPI, and 2D STEAM methods were compared to those obtained from the 2D DAM method. Although the 2D DAM method does not constitute a true gold standard, the results from this comparison were used to assess the accuracy of the methods under study.

## MATERIALS AND METHODS

Data were acquired on eight healthy volunteers (seven males, mean age 34 years), using a 3T whole-body Magnetom TIM Trio (Siemens Healthcare, Erlangen, Germany), operated with a body transmit coil and a 12-channel head-only receive coil. The study was approved by the local ethics committee, and written informed consent was obtained from the subjects prior to scanning. Four successive acquisitions of the 3D AFI, 3D EPI, and 2D STEAM methods were recorded on each subject within one scanning session in order to assess the reproducibility of these methods. The image resolution was 4 × 4 × 4 mm^3^ for all three methods. Due to its long acquisition time, the reference DAM scan was acquired only once on each subject, with an image resolution of 3 × 3 × 10 mm^3^.

### 3D AFI Method

Image acquisition was implemented with the following parameters: matrix size = 64 × 60 × 48, field of view = 256 × 240 × 192 mm^3^, echo time (TE) = 3.13 ms. A Hamming-filtered sinc RF pulse with a nominal time-bandwidth product of 1 was used for spin excitation, with a duration of 500 μs and nominal flip angle α = 60°. Spoiling of the magnetization was implemented using the modified spoiling scheme by Nehrke (25), with a phase increment of ϖ = 129.3° and spoiler gradients with an amplitude of 26 mT/m and durations of 1/3 ms for TR_1_/TR_2_, based on simulation results of RF spoiling in FLASH acquisitions similar to simulations described in Preibisch and Deichmann (29). Diffusion weighting with reduced sensitivity to physiological artifacts was achieved by “three-lobes” flow-compensated gradient pulses with an amplitude of 26 mT/m and duration of 10.5 ms (with relative durations 1:2:1) (30). This corresponds to a diffusion damping of d ∼ 6.10^−2^, assuming a diffusion coefficient D = 8.10^−4^ mm^2^ sec^−1^ and neglecting the contribution of higher-order coherence pathways (25,31). We set TR_1_ to 50 ms, the minimum achievable value due to the duration of the flow-compensated gradients. We set TR_2_ to 150 ms in order to keep the acquisition time below 5 min. Parallel imaging (GRAPPA reconstruction, acceleration factor 3, 18 reference lines) was used in the phase direction, and partial Fourier (factor 6/8) was applied in the partition direction, yielding a total acquisition time of 4 min 12 sec.

### 3D EPI Method

Image acquisition was implemented with the following parameters: matrix size = 64 × 48 × 48, field of view = 256 × 192 × 192 mm^3^ (17% oversampling along the partition direction), TE_SE_/TE_STE_/TM/TR = 33.2/66.73/33.53/500 ms. A Hamming-filtered sinc RF pulse with nominal time-bandwidth product of 12 was used for spin excitation, with a duration of 4ms and nominal flip angle α = 90°. The duration of the rectangular SE and STE pulses was set to 700 μs. The respective flip angles for the SE/STE pulses were varied between 160°/80° and 200°/100° in steps of 10°/5°. The total experimental time was 2 min 20 sec. An additional *B*_0_ map was acquired on each subject, as described in Weiskopf et al. (32), adding an extra 2 min of scan time. In the postprocessing of the 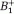 maps, voxels whose fit exhibited more than 5% error and/or where the local *B*_0_ deviation exceeded 110 Hz were replaced using the padding procedure described in the Theory section.

### 2D STEAM Method

Image acquisition was implemented with the following parameters: matrix size = 64 × 48, field of view = 256 × 192 mm^3^, TE/TM/TR = 6/30/24103 ms. Fifty-six slices were selected, with a thickness of 2.9 mm and a distance factor of 38%, resulting in a gap of ∼1.1 mm. Hamming-filtered sinc RF pulses with a time-bandwidth product of 2 and duration of 1280 μs were used for spin excitation and “flip-back”. A shorter duration of 800 μs was used for signal readout. The nominal flip angles were kept constant at 90° and 12° for the excitation and readout pulses, respectively, and set to α_1_/α_2_ = 60°/100° for the “flip-back” pulse. The acquisition time for one volume was 24 sec. Three successive volumes were acquired for averaging, leading to an overall acquisition time of 2 min 24 sec per averaged map. Calibration values α_max_ = 1.5755 and q = 0.6688 were used in the following analyses. The deviation of these values from those of the original calibration method is attributed to differences in pulse shape of the “flip-back” pulses (18).

### Reference DAM Method

Image acquisition was implemented with the following parameters: gradient-echo EPI readout with 12 slices, slice thickness/gap = 5 mm/5 mm, matrix size = 64 × 64, field of view = 192 × 192 mm^3^, effective resolution of 3 × 3 × 10 mm^3^, TE = 25 ms, and TR = 25 sec to allow for complete relaxation of the magnetization between the scans. Rectangular RF pulses were used for presaturation of the magnetization with a duration of 500 μs. The nominal flip angle values of the presaturation pulse were α_1_/α_2_ = 22°/66°, separated by a factor 3 in order to increase the signal difference between the two images and enhance the signal-to-noise ratios in the resulting 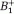 map. Spin excitation was implemented using slice-selective sinc RF pulses with a duration of 2560 μs (nominal flip angle value: α = 90°). The total acquisition time was 10 min.

### Data Analysis

All data processing was performed using MatLab (The MathWorks Inc., Natick, MA), version 7. This included the use of SPM8 (http://www.fil.ion.ucl.ac.uk) for data conversion, brain extraction, coregistration, and reslicing of the individual 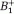 maps. *B*_0_ maps were processed with a modified version of the SPM fieldmap toolbox (28). Custom-made MatLab scripts were used for estimation of the 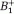 maps from the acquired datasets and for data analysis.

Flip angle values in all 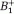 maps were calculated as a percentage of the nominal flip angle (in percentage units = p.u.). This allowed for straightforward comparison of the results between the methods, assuming a linear relationship between nominal and local flip angles. 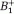 maps were smoothed with an 8 mm full-width-at-half-maximum gaussian kernel prior to further analysis. For each 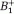 mapping method, the voxelwise mean and standard deviation (SD) across the four repeated measurements were calculated. The map of differences between the voxelwise mean for each technique and the DAM reference values was used to determine the accuracy or bias of each method. The SD maps were used as indicators of reproducibility or precision.

To quantitatively compare the different methods, the voxelwise means, standard deviations, and differences from the reference were averaged over an ROI in the central part of the brain, providing values *B*_1_*-mean*, *B*_1_*-SD* and *B*_1_*-bias*, respectively, for each technique and each subject. The ROI was created on an individual basis using the same automated procedure for all subjects: the short TE magnitude *T*_1_-weighted image acquired as part of the *B*_0_ map acquisition was segmented into tissue probability maps (33). Cerebrospinal fluid, gray matter, and white matter tissue segments were then combined to form a brain mask. Morphologic operators were used to erode the mask by 10 voxels followed by smoothing and thresholding to create a region located well inside the brain. Repeated-measures analysis of variance (ANOVA) (SPSS, version 17.0; IBM Company, New York, NY) of *B*_1_-mean and *B*_1_-SD was used to test for significant differences between the sequences. Standard post hoc paired *t* tests were performed to explore any difference further. Statistical significance was assessed at a threshold of *P* < 0.05.

## RESULTS

**FIG. 2:**
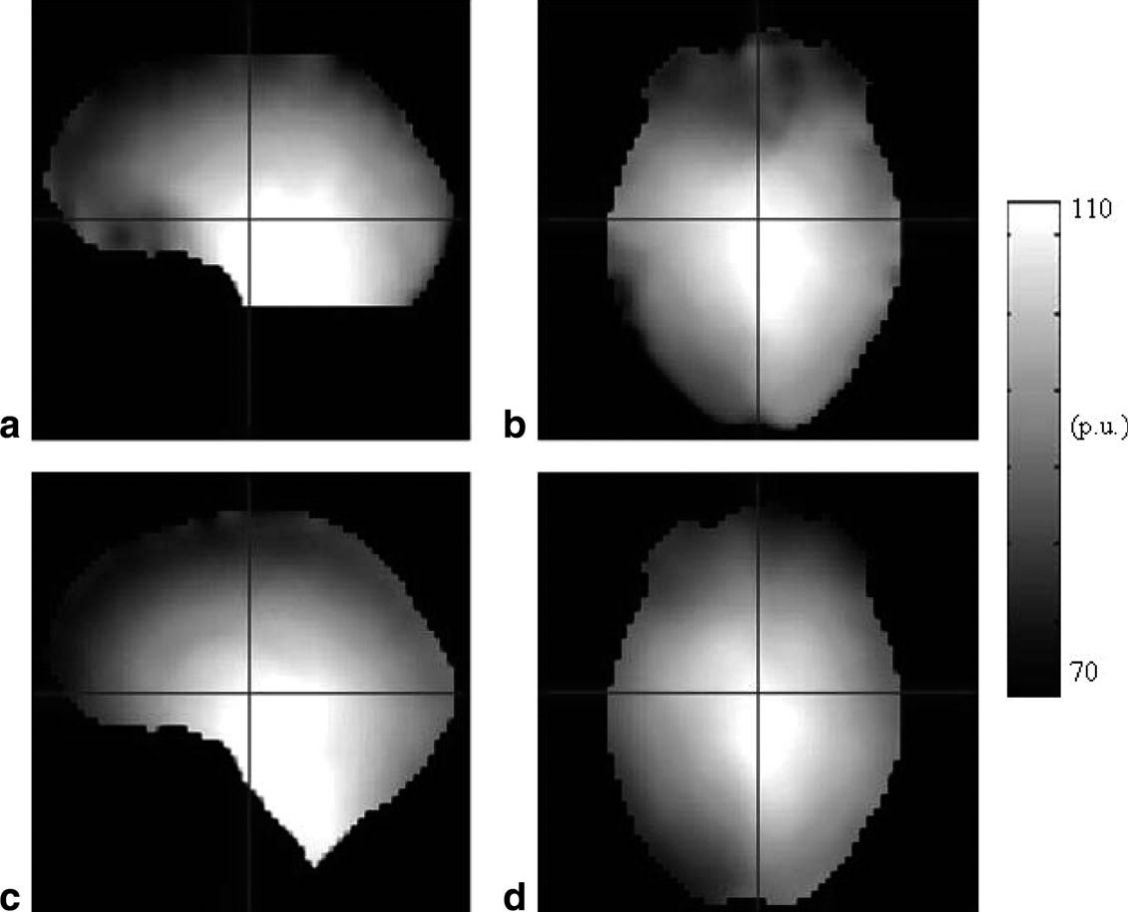
Sagittal and axial views of *B*_1_^+^ maps for a single subject acquired using the DAM **(a,b)** and 3D AFI **(c,d)** methods. Note the susceptibility-induced artifacts in the orbitofrontal cortex in the DAM method; 184 × 152 mm (600 × 600 DPI).

**FIG. 3:**
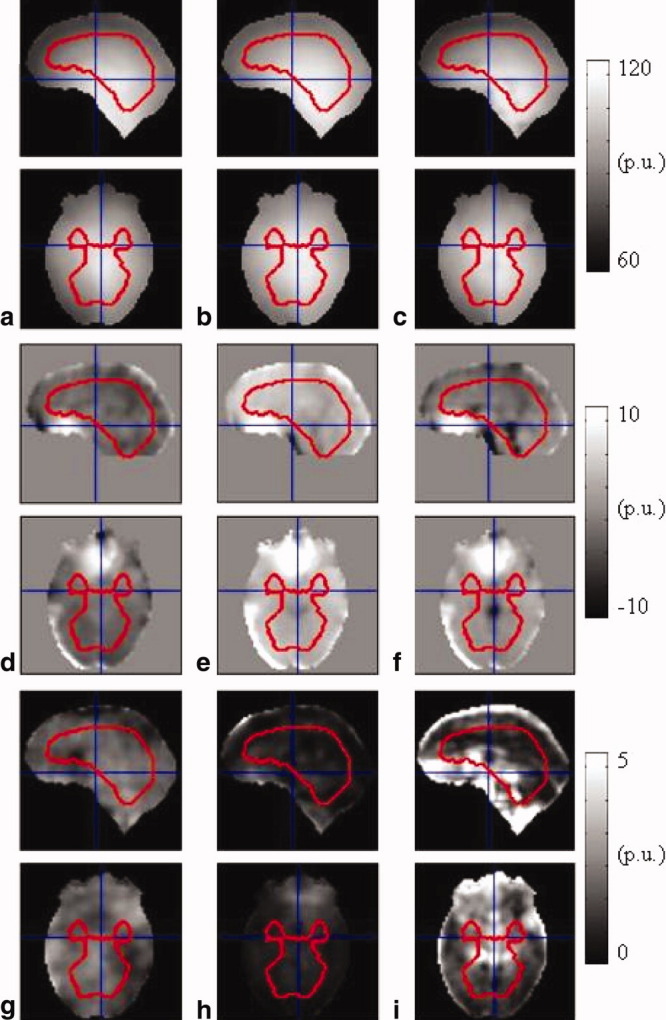
Sagittal and axial views for a single subject for the 3D AFI **(a,d,g)**, 3D EPI **(b,e,h)**, and 2D STEAM **(c,f,i)** *B*_1_^+^ mapping methods. **a–c**: *B*_1_^+^ maps obtained using each of the methods; **(d–f)** difference images with the reference DAM method; **(g–i)** standard deviation maps taken over four successive repetitions of each method. The red outlines illustrate the ROI used in our study; 164 × 254 mm (600 × 600 DPI).

Figure 2a-d shows sagittal and axial views of typical 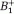 uniformity patterns acquired using the DAM (Fig. 2a,b) and 3D AFI (Fig. 2c,d) methods for a single subject. Local flip angles vary between ∼70 p.u. and 120 p.u. of the nominal value over the brain volume. Note that little “shine-through” of brain structure into the 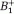 maps (an indication of inaccurate 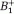 values) is apparent even around the ventricles, reflecting the low sensitivity of these methods to the long *T*_2_ times of cerebrospinal fluid and to flow effects. A diagonal pattern of 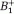 nonuniformity is present in the axial views. This effect, detected by all methods presented here, is due to the interaction between the subject and the 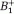 field (34). In the DAM 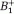 maps, off-resonance artifacts can be seen in the region of the orbitofrontal cortex.

Figure 3a-c shows sagittal and axial slices through typical voxelwise mean 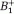 maps acquired using each method under study. For the 3D AFI and 3D EPI methods, only smooth variations are present and no “shine-through” of brain structure is apparent. On the 2D STEAM 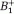 maps, the latter artifact is visible at the level of the third ventricle due to flow effects. Voxelwise difference images between the methods under study and the reference method are shown in Fig. 3d-f. Overall, the 3D EPI method overestimates the flip angles compared to the reference method, whereas the 3D AFI method underestimates them. All difference maps show “shine-through” of the ventricles of amplitude ∼1 p.u. Large difference values are visible for all methods in the orbitofrontal cortex. Both effects arise from artifacts in the reference DAM method, as shown in Fig. 2a,b, and are not apparent in pairwise comparisons of the three methods (not shown). Artifacts are also visible in the outer parts of the brain in all difference images due to partial-volume effects arising from the large slice thickness used for the reference method. Figure 3g-i shows the voxelwise standard deviations of the 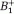 maps obtained from four repetitions of each of the methods. While the 3D EPI method was most stable (SD <1.5 p.u.), significant instabilities were found for the 2D STEAM method (SD >5 p.u.), particularly at the location of the ventricles and at the base of the brain where high blood flow and pulsating cerebrospinal fluid flow are present. The results shown in Fig. 3 were representative of all subjects.

A quantitative comparison across subjects was performed by averaging the 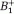 results over an ROI covering the central regions of the brain, where the reference DAM method is expected to perform optimally. This ROI is illustrated by the red contour in Fig. 3. The means and standard deviations of *B*_1_-mean values across subjects were 99.3 ± 1.7, 104.7 ± 1.6, 101.9 ± 2.1, and 100.7 ± 1.6 p.u. for the 3D AFI, 3D EPI, 2D STEAM, and reference methods, respectively. Repeated measures ANOVA showed a dependence of *B*_1_-mean on the 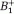 mapping method (F = 84, df = (3,21), *P* < 0.001), although these differences were relatively small and did not exceed 5 p.u. Post hoc paired *t* tests showed statistically significant differences between all methods (t >5.9, df = 7, *P* < 0.001), except for 2D STEAM versus 2D DAM (t ≈ 2.3, df = 7, *P* > 0.05).

**FIG. 4:**
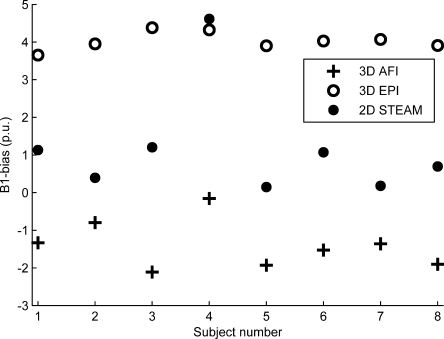
Bias/accuracy. Flip angle differences between each of the methods under study and the reference DAM technique averaged over the ROI (*B*_1_-bias); 103 × 71 mm (600 × 600 DPI).

Figure 4 shows the *B*_1_-bias values for each method and each subject, providing a measure of accuracy of the methods. The means and standard deviations of *B*_1_-bias across subjects were −1.4 ± 0.7 p.u., 4.0 ± 0.2 p.u., and 1.2 ± 1.5 p.u. for the 3D AFI, 3D EPI, and 2D STEAM methods, respectively. An outlier subject in the 2D STEAM dataset (#4) led to a wider range of *B*_1_-mean values. Omitting the outlier subject in the 2D STEAM dataset decreased the corresponding mean *B*_1_-bias to 0.7 ± 0.5 p.u.

**FIG. 5:**
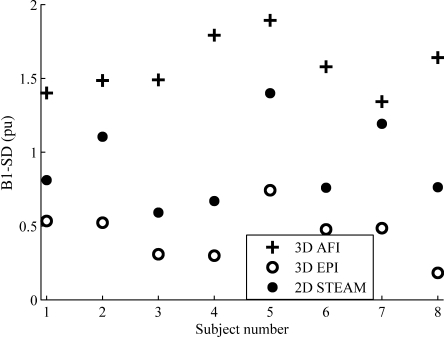
Reproducibility/precision. Standard deviation of four successive repetitions of each *B*_1_^+^ mapping method averaged over the ROI (*B*_1_-SD); 103 × 72 mm (600 × 600 DPI).

Figure 5 shows *B*_1_-SD for each method and each subject, providing a measure of the reproducibility/precision of the methods. The means and standard deviations of *B*_1_-SD values across subjects were 1.6 ± 0.2, 0.4 ± 0.2, and 0.9 ± 0.3 p.u. for the 3D AFI, 3D EPI, and 2D STEAM methods, respectively. The standard error for each mean *B*_1_-SD value is in good agreement with the SD of each *B*_1_-bias value (i.e., comparing Figs. 4 and 5), which shows the coherence of our results. Repeated-measures ANOVA tests showed a dependence of *B*_1_-SD on the 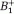 mapping methods (F = 80, df = (2,14), *P* < 0.001). Post hoc paired *t* tests showed statistically significant differences between all *B*_1_-SD distributions (t > 5.9, df = 7, *P* < 0.001).

## DISCUSSION

We optimized and compared three published methods for rapid mapping of the 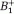 field in the human head at 3T: 3D AFI (22), 3D EPI (23), and 2D STEAM (18). In order to assess the accuracy of the techniques under study, results were compared to the 2D DAM method (24), chosen as a reference in the experiment design. Before optimization, each 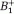 mapping method showed specific artifacts that were addressed in this study. After optimization, results were found within 5 p.u. agreement with the reference 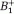 maps. Standard deviations over repetitions were below 2 p.u. The overall errors were therefore found small compared to the typical variations of ∼50 p.u. observed at 3T across the brain.

### 3D AFI Method

The signal equation for the dual-excitation FLASH experiment used in 3D AFI does not account for residual transverse coherences (29). Reducing these by choice of small flip angles is not an option, because the separation of flip angle bias and relaxation becomes ill conditioned, as shown by the rational approximation of the steady-state signals (11,35). For the large flip angle (60°) used here, the addition of flow-compensating diffusion damping gradients reduced bias due to incomplete spoiling of transverse coherences while minimizing the flow and motion sensitivity of the method, an approach similar to the one used for steady-state free-precession sequences in Ding et al. (36).

Sufficient diffusion spoiling required long gradient pulses and therefore longer TR values (50/150 ms), resulting in an increased acquisition time. As a result of the increased TR values, misestimation of the flip angles from longitudinal relaxation effects may reach up to ∼1 p.u. locally, according to simulations similar to those by Yarnykh (22). Further spoiling of the transverse coherences would require longer diffusion gradients since the spoiler amplitudes cannot be significantly increased further on a typical whole-body clinical scanner. This would result in longer TR values and an increased acquisition time and flip angle bias due to longitudinal relaxation effects.

### 3D EPI Method

Distortion correction mitigated spatial distortions of the 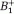 maps obtained using the 3D EPI method by two voxels or more in areas such as the inferior temporal lobe and orbitofrontal cortex. The padding procedure (RMS and *B*_0_) corrected 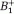 in 3.9 ± 0.7% of the voxels in the flip angle maps by an amount of 3.4 ± 0.8 p.u. averaged across subjects. The brain regions affected by RMS-padding were mainly near the superior sagittal sinus and the circle of Willis, where large blood vessels are present (∼ 3.8% of the brain volume). Brain vasculature may therefore be the main contributor to these errors on the flip angle estimates. *B*_0_-padding only affected the orbitofrontal cortex due to the high susceptibility gradients in this region (∼0.6% of the brain volume). If applied on areas of larger size, padding might fail to reflect accurately the topological features of the true 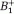 field. The threshold for *B*_0_ padding depends on the SE/STE RF pulses used experimentally and may need to be recalibrated if these are changed.

The 3D EPI method showed the most reproducible results over repetitions and across subjects but the largest systematic bias compared to the reference DAM method. The origin of this systematic bias remains unclear. According to Jiru and Klose (23), the effect of longitudinal relaxation during the mixing time may not exceed ∼0.5 p.u. for the range of flip angles present within the ROI. Based on preliminary experiments on the effect of off-resonance on the 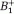 maps, we found that only 3 ± 1% of the voxels within the ROI were biased by more than 0.5 p.u. due to off-resonance, averaged across subjects. For these voxels, the average off-resonance bias was 0.65 ± 0.07 p.u., small compared to the ∼4 p.u. systematic bias observed over the ROI.

The precision of the method was largely determined by the number of nominal flip-angle values (23). However, additional acquisitions seemed unnecessary, since the current implementation already achieved the highest precision of all methods. For the data presented here, setting the threshold for RMS padding to 5 p.u. proved to be in general a good tradeoff between denoising the data sufficiently and minimal reduction of degrees of freedom in the dataset.

### 2D STEAM Method

For the 2D STEAM method, calibration of the nonlinearities of the slice-selective pulse was improved by cross-calibrating with an independent (AFI) 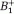 mapping method. The calibration was implemented on the entire volume of a gel phantom in order to obtain more reliable results across the whole range of flip angles and reduced sensitivity to fitting errors in the reference curve. The results of the calibration were used as a multiplicative factor of the local flip angle (18), removing any systematic bias between the two methods. As a result, a similar level of accuracy between the two methods might be expected on human subjects. The high time efficiency of the 2D STEAM method permitted averaging over three 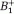 maps based on successive acquisitions, yielding an averaged 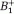 map with a reduced level of instability.

Despite averaging, local instabilities beyond 5 p.u. persisted due to physiology-related artifacts. Averaging over a higher number of acquisitions might be desirable as the total acquisition time for three repetitions was still shorter than for the other methods (∼2.5 min instead of ∼4.5 min). Averaging of image intensities obtained for each value of the flip-back pulse instead of averaging the 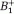 maps (as done here) may reduce instabilities further. Reduction of in-flow artifacts may alternatively be achieved by physiologic triggering. Fitting a sine curve to six different flip-back angles (37) may reduce the sensitivity of the method to spurious artifacts, simplify the calibration, and allow the use of a padding procedure similar to that of the 3D EPI method. Here, the original method and the quadratic approximation of the flip angle dependence were retained to enable the comparison with the other two-point methods. The presence of an outlier subject (#4) in the STEAM dataset for which higher flip angles were measured consistently across repetitions remains unexplained.

### Comparison of the Methods

The main findings of this comparison study are su mmarized in Table 1. All optimized methods provided 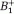 maps with errors less than 5 p.u. for a total scan time of <4.5 min. The 3D EPI method showed the highest reproducibility with *B*_1_-SD <0.7 p.u. The intrasubject SD of the 2D STEAM varied significantly across the brain due to its sensitivity to flow. The 3D AFI method had the lowest reproducibility (*B*_1_-SD <1.9 p.u.), but high compared to the 50 p.u. variations of the flip angles across the brain. Deviations from the DAM reference method were found between 1 and 2 p.u. for the 3D AFI and 2D STEAM methods. The STEAM cross-calibration method implemented here likely contributed to the similar accuracy found for both methods. The accuracy of the 3D EPI method was lower, with ∼4 p.u. deviation from the reference. If necessary, this bias could be reduced by cross-calibration with a reference 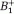 mapping method, similar to the calibration performed for the 2D STEAM method.

**Table 1:** Summary of Characteristics for the Three 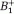 Mapping Methods Presented in This Study

	3D AFI	3D EPI	2D STEAM
B_1_-bias (p.u.)	−1.4 ± 0.7	4.0 ± 0.2	1.2 ± 1.5
B1-SD (p.u.)	1.6 ± 0.2	0.4 ± 0.2	0.9 ± 0.3
Calibration	No	No	Yes (of slice-selective pulse)
Postprocessing	No	Yes (correction of undistortions and off-resonance effects)	Yes (reduction of physiological noise)
Processing time^a^	<5 sec	<2 min	<5 sec
Acquisition time	4 min 12 sec	2 min 20 sec + 2 min (*B*_0_ map)	2 min 24 sec

a PC, Intel Xeon, 3.2 GHz, eight cores, 12-GB random-access memory (Dell Inc., Round Rock, TX).

In principle, the 2D STEAM method is the fastest and can generate a 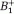 map in only 2 × 24 sec. The averaging of three acquisitions to reduce in-flow effects brought the total scan time to about 2.5 min. Despite averaging, this method remained faster than the 3D AFI method that had a scan time of ∼4.5 min, even with 3-fold GRAPPA acceleration and partial Fourier sampling. The scan time for the 3D EPI was approximately 2.5 min. For this method, implementation of parallel imaging along the phase and partition directions would significantly reduce the acquisition time and the amount of spatial distortions in the EPI images. An additional *B*_0_ map was necessary for correction of off-resonance artifacts, adding another ∼2 min to the overall scan time. Since *B*_0_ maps are frequently acquired for different purposes in MRI examinations, this may often not be an additional overhead. Furthermore the *B*_0_ mapping can be made faster, since large coverage (beyond the 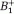 map) and rather long TRs were used.

The implementation of the 3D AFI method was straightforward, with minimal postprocessing and no need for calibration. In contrast, the 3D EPI method required postprocessing steps, including unwarping, masking, and padding of the 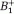 maps. The 2D STEAM method relied on an extra (one-time) calibration for nonlinearities due to the slice-selective RF pulses to maximize its accuracy but otherwise required minimal postprocessing.

The 3D AFI method required comparably higher gradient power than the 3D EPI and 2D STEAM methods since the flow-compensated spoilers were driven with high amplitudes and duty cycle to keep the TR minimal. This puts high demand on the scanner hardware and may also impact on subjects' compliance due to mechanical movement (similar to diffusion imaging).

### Considerations

The 2D DAM method is conceptually simple and insensitive to longitudinal relaxation times and slice-selection nonlinearities. Therefore, it was chosen as a reference in order to assess the accuracy of the 3D AFI, 3D EPI, and 2D STEAM methods despite its low resolution, long acquisition time, and sensitivity to off-resonance effects. While an ROI covering the entire brain would enable a more complete comparison between the methods, we focused on an ROI inside the brain and covering most of the brain volume, where the reference DAM method was seen to perform optimally. As a result, the impact of physiologic noise sources present in outer brain regions (e.g., blood flow) was excluded from the study.

Fast whole-brain 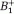 mapping methods as presented here are currently not readily available on clinical MR systems and still need to be implemented using pulse-sequence programming on site. All methods were implemented here on a system with a high-power RF amplifier (35 kW) and gradient system (slew rate up to 200 mT/m/ms (38)). The sequence parameters may need to be adapted when implemented on another system, possibly affecting the quality of the results. In particular, the RF pulse duration should be minimal to reduce off-resonance sensitivity while the RF amplifier should still operate in the linear regime. For the 3D sequences, Hamming-filtered sinc pulses were used for slab-selective excitation in order to achieve an optimal slice profile. A sufficient slab thickness might be desirable in order to minimize inflow effects, and oversampling in the partition-encoding direction should be used to reduce potential wraparound. High gradient power enables fast EPI readouts, reducing geometric distortions and the mixing/echo times in the 3D EPI method. It also allows for efficient spoiling in the 3D AFI method while minimizing TR. Relaxation times and off-resonance effects should be considered while implementing the methods at different *B*_0_ field strengths. Also, the use of head transmit RF coils would increase in-flow effects compared to the body transmit coil used here since the nonselective RF pulses used for determining the local transmit field would saturate flowing blood outside the brain less efficiently (39). Also, localized surface coils may enhance signal from the scalp that can increase the amplitude of motion artifacts (40).

Although all 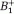 mapping methods were optimized and validated for brain imaging, they may be used to scan other organs. For example, the intrinsic robustness of the 3D AFI method against susceptibility-related distortions makes it a good candidate for 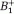 mapping in areas affected by susceptibility artifacts (e.g., near bones), which might be challenging for the 3D EPI method. Large vessels and blood flow (e.g., in the abdomen and thorax) are expected to exacerbate the flow artifacts in the 2D STEAM method.

## CONCLUSION

The accuracy and precision of three 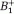 mapping methods—3D AFI, 3D EPI, and 2D STEAM—were assessed in the context of human brain imaging at 3T. The acquisition time for all three methods was below 5 min. For each method, the observed artifacts were mainly due to the specific sequence used for spatial encoding. Optimization of these methods yielded overall errors less than 5 p.u. in the central regions of the brain, small compared to the typical variations of ∼50 p.u. in the 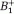 field observed across the brain at 3T. The choice of a particular method for routine 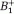 mapping may depend on the properties of the 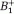 maps required by the user. If high reproducibility is desired, the 3D EPI method is recommended. However, postprocessing is required for this method in order to correct for image distortions and flip-angle bias. The intrinsic robustness of the 3D AFI method against susceptibility-related distortions makes it a good candidate for 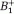 mapping in areas affected by susceptibility artifacts and at higher fields. However, it poses rather high demands on the scanner hardware and requires a relatively long minimal scan time. If rapidity is of primary importance, the 2D STEAM method might be preferred. However, this method is most sensitive to physiologic artifacts and requires calibration using an independent method.
